# Physician’s Visiting List, for 1863

**Published:** 1862-10

**Authors:** 


PHYSICIAN'S VISITING LIST, FOR 1863. Published by LINDSAY & BLAKESTON, of Philadelphia.

Its contents are: an Almanac, a table of Signs, Marshall Hall's ready method in Asphyxia, Poisons and their Antidotes, Table for calculating the period of Utero-Gestation, Blank leaves for visiting lists, Memoranda, Addresses of Patients and others, Accounts asked for, Memoranda of Wants, Obstetric engagements, Vaccination engagements, &c.

